# BMCGM: A Behavior Economics-Based Message Transmission Cooperation Guarantee Mechanism in Vehicular Ad-hoc NETworks

**DOI:** 10.3390/s18103316

**Published:** 2018-10-03

**Authors:** Jiaqi Liu, Nan Zhong, Deng Li, Hui Liu

**Affiliations:** 1 School of Software, Central South University, Changsha 410075, China; liujiaqi@csu.edu.cn (J.L.); neilzhong@csu.edu.cn (N.Z.); 2 School of Information Science and Engineering, Central South University, Changsha 410075, China; 3 Missouri State University Computer Science, 901 S. National Ave, Springfield, MO 65897, USA; huiliu@missouristate.edu

**Keywords:** VANETs, behavior economics, reciprocal altruistic, network formation game, cooperative communication

## Abstract

Vehicular Ad-hoc NETwork (VANET) is a special mobile ad hoc network that composed of facilities such as vehicle nodes and roadside units. Message transfer among vehicle nodes has been a great challenge due to the network’s highly variable topology and the selfish nature of vehicle nodes. Thus, it is very necessary to propose a mechanism to improve the cooperation among vehicle nodes to guarantee the effective message transmission. Currently, incentive-based cooperation mechanisms are commonly used to encourage nodes to participate in message transmission. Those mechanisms are based on traditional economics and generally assume that the decision-making behavior of nodes is completely independent. Also, the cooperation of nodes depends on whether the cooperation behavior can obtain the higher utility. But researches in behavioral economics have shown that due to the existence of altruistic reciprocity, the behavior of nodes is affected by not only their utility but also the behavioral motives of other nodes, so as to obtain different results from traditional incentive-based mechanisms. Therefore, the paper introduces the reciprocal altruistic from behavioral economics and proposes the reciprocal altruistic factor to reconstruct the utility function of nodes. The reconstructed utility function reflects the interaction of behavioral motives among nodes, which promotes the node’s cooperative behavior. Also, since the Network Formation Game (NFG) is a common mathematical model for studying the interaction and communication links formation among network nodes, hence the paper regards NFG in traditional economics as the research object. A Behavior Economics-based Message Transmission Cooperation Guarantee Mechanism named BMCGM is proposed, which motivates nodes to participate in the message transmission to reduce the transmission delay ratio. The simulation results show that the BMCGM reduces message transmission delay by at least 30.3% compared with the recent representative cooperation transmission mechanism.

## 1. Introduction

VANET is a special mobile ad hoc network (MANET) that has special characteristics compared with mobile ad hoc networks such as highly dynamic topologies, frequently disconnected links, restricted moving directions (which effected by the direction of the road, traffic lights, etc.) and without restrictions for energy and storage [1,2]. Vehicles in VANET can communicate with other vehicles via the On-Board Unit (OBU) and can also communicate with the RoadSide Unit (RSU) via the OBU [3].

While VANET provides convenient services [4,5,6,7,8,9], the nodes need to participate in the message transmission actively. However, when nodes participate in cooperation, they will cost their own communication resources. At the same time, there are some security or privacy issues such as leakage of location information. Above factors may reduce the willingness of nodes to cooperate. Thus, how to effectively promote node’s participation in message transmission is one of the research hotspots of current vehicle networks.

Current cooperation mechanisms in VANET can be divided into five aspects: the incentive-based, the punishment-based, the retrieval-based, the misbehavior detection-based and the mobile social networking-based cooperation mechanism. Among them, the incentive-based and the retrieval-based are widely studied.

Some current incentive-based cooperation mechanisms [10,11,12,13,14] are generally based on traditional economics. Most of them assume that nodes are rational and the behavior of nodes is not related to other nodes’ decisions. The utility of nodes depends on some aspects such as the success rate and the consumption of message transmission. On the other hand, in order to maximize own utility, nodes always choose to cooperate under this condition. However, some researches in behavioral economics show that [15], because of the existence of altruistic reciprocity, the behavioral decision of nodes considers not only the economic utility but also the behavior of other nodes. The actual perceived utility affects the behavioral choice of the node.

The NFG is a common mathematical model for studying the interaction and communication links formation among network nodes, thus the paper regards NFG in traditional economics as the research object. We construct a cooperative willingness function based on reciprocal altruistic. It can reflect whether other nodes are friendly or unfriendly. Then we design a reciprocal utility function based on cooperative willingness function. The utility of a node is determined by the combination of economical utility and reciprocal utility. We analyze the nodes’ behavior based on reciprocal altruistic and propose BMCGM to promote cooperation behavior. Our contributions are listed as follows:

1. A reciprocal altruistic factor is proposed to build the actual perceived utility of the nodes, which overturns the existing definition of the utility function and increases the cooperation rate of nodes by reconstructing the utility function;
2. We take NFG in traditional economics as the research object and analyze the decision-making behavior of nodes while considering the reciprocal altruistic of nodes. A new message transmission cooperation mechanism named BMCGM is proposed to reduce the message transmission delay.

The rest of this paper is organized as follows. Section 2 discusses the related works. Section 3 presents the system model; the system model is the basis of BMCGM proposed in this paper. Section 4 provides a detailed description on the BMCGM. Section 5 compares BMCGM with the existing cooperation mechanism through simulation experiments. Section 6 concludes the paper.

## 2. Related Work

In this section, we briefly review related works on cooperation guarantee mechanisms and reciprocal altruistic.

### 2.1. Cooperation Guarantee Mechanisms

According to Reference [16], the current message cooperation guarantee mechanism in VANETs can be divided into five aspects.

Punishment-based cooperative guarantee mechanism punishes nodes that make malicious or selfish behavior in the network. Jesudoss et al. [17] proposed a punishment-based cooperation guarantee mechanism, which gives corresponding punishment to the nodes that refused to cooperate in the cluster.

The cooperation guarantee mechanism based on malicious behavior detection detects nodes with selfish and malicious behavior and expels them from the network. An anonymous credential system was proposed in Reference [18] to detect and limit the frequent replacement of credentials by malicious nodes and to revoke the credentials of malicious nodes.

When it comes to the cooperation guarantee mechanism in mobile social networks, the social relationships that exist among the nodes are explored and used to promote the participation of the nodes. In Reference [19], a Vehicular Social Network Protocol (VSNP) was proposed to promote cooperation among nodes.

The current mainstream cooperation guarantee mechanisms are the incentive-based and the reputation-based, which are described in detail as follows.

A. Incentive-based cooperation transmission mechanisms

The incentive-based cooperation transmission mechanism provides incentives or rewards to the nodes to promote cooperation among nodes. In order to promote the message transmission among nodes in VANETs, researchers proposed some message transmission cooperation guarantee mechanisms based on utility maximization. In Reference [11], the authors proposed a service-based Cooperation Message transmission model (COMES). In COMES, the node decides whether to stay in the coalition or to leave the alliance according to the obtained incentives, which are determined by factors such as the average message receiving rate of the coalition. A secure cooperative download incentive mechanism (SIRC) was proposed in Reference [12]. While ensuring secure transmission, SIRC minimizes the payment risk of the destination node. The nodes participating in the message forwarding can only obtain all the rewards after being confirmed by the destination node. Xu et al. [13] proposed an incentive-based advertising message transmission mechanism. It provides the same reward for all nodes which involved in the transmission and does not consider the different transmission costs generated by each node. A coalition formation game-based cooperation mechanism was proposed in Reference [14], which discusses the rewards of the source node and the relay node. The value of the rewards is determined by parameters such as the number of participating cooperation messages and the time of carrying the message. Jesudoss et al. [17] proposed a cooperation mechanism with rewards and punishments. In this mechanism, trusted nodes receive rewards in the cluster, which is related to the consumption of participation. A Rewards and Bounds-based cooperation transmission mechanism named RBI was proposed in Reference [20], which constructs a forwarding tree from the source node to the destination node. RBI provides rewards to nodes which participate in message transmission based on their effort degree. Additional bonus is provided to the last two nodes which are finally involved in forwarding. The rewards received by the nodes are determined by factors such as the number of transmission messages and Time To Live (TTL). In Reference [21], the authors proposed an incentive-based collaborative content download mechanism, the incentives obtained by the nodes are determined by the combination of rewards and consumption to transmit the content. In Reference [22], the authors studied a network coding-based message forwarding mechanism and constructed an incentive matrix to motivate relay nodes to forward message. The node’s incentive value depends on the consumption of encoding and forwarding messages. NFG-based Cooperation Transmission Mechanism Algorithm (NGOMA), was proposed in Reference [23]. The mechanism is based on the NFG [24], which refers to a network game involving a certain number of independent decision makers (participants). The decision makers independently make decisions and interact to form a suitable connected graph to connect them. And the goals and the benefits that the decision makers received in the graph will affect the resulting network directed graph. NGOMA improves the message transmission mechanism. When the link from the source node to the destination node fails, the surrounding neighbor nodes calculate an incentive function and retransmit the message to the destination node according to the incentive value. Since NGOMA is currently a representative cooperation guarantee mechanism in VANET, we use NGOMA for comparison in order to evaluate the BMCGM.

B. Reputation-based cooperation transmission mechanisms

In the reputation-based cooperation transmission mechanism, nodes which have contributed to their network and have good reputation can use network resources, while those with poor reputation will be evicted from the network. Hu et al. [25] proposed PTRS, which uses the Dirichlet distribution to calculate the feedback reputation to determine the credibility of the node. The authors in Reference [26] proposed a reputation-based mechanism, where a reputation system is designed for the platform nodes to collect feedback from surrounding nodes and determine the reputation of the nodes. A message reliability-based reputation mechanism was proposed in Reference [27]. The reliability of the message is related to the reputation value of nodes which transmitted this message. Li et al. [28] proposed a reputation mechanism which measures the reliability of the message by collecting feedback reputation. A local and recommended-based reputation mechanism was proposed in Reference [29], which introduces Dempster-Shafer Theory to update local reputation.

In punishment-based and misbehavior detection-based cooperative guarantee mechanisms, such as [17,18], the malicious node needs to be detected precisely. Therefore, the core nodes need to collect the communication information of surrounding nodes. They should have high storage capacity and high processing capability, which makes these mechanisms unsuitable for the general situation.

The above-mentioned incentive-based and reputation-based mechanisms are mostly based on the expected utility theory of traditional economics, such as mechanisms proposed in Reference [11,12,13,14,20,21,22,23]. Those mechanisms assumed that nodes are independent of each other and each decision behavior of a node depends on the utility value itself and does not affected by the decision motivation of other nodes. The reputation-based cooperation transmission mechanisms proposed in Reference [25,26,27,28,29] assumes that nodes tend to cooperate with the nodes which have high reputation values. The value of reputation becomes the sole motivation for node’s behavior decision and does not consider the behavioral motives of other nodes. In recent years, more and more research results in behavioral economics [30,31,32] showed that the decision-making motivations of other nodes have an intrinsic influence on them due to the reciprocal altruistic. The incentive function based on the maximization of economic utility ignores the influence of this internal factor, thus the effectiveness of cooperation promoting of these mechanisms is under determined. Therefore, this paper uses the reciprocal altruistic from behavioral economics to design the utility function of nodes and analyzes the influence of reciprocal altruistic on the behavior decision and utility of nodes and hence obtains a new message cooperation transmission mechanism named BMCGM.

### 2.2. Reciprocal Altruistic

Reciprocal altruistic is a kind of help to others at the expense of their own interests. While giving help, they also look forward to the help of others in the future. They give rewards to the motives of good intentions and punish malicious motives.

In the field of economics, researchers mainly study reciprocal altruistic in terms of economics behavior and game and assume that participants in the game will reward goodwill behavior and punish malicious behavior. Stanca and Falk et al. [33,34] considered the individual’s behavioral motives and believed that the individual’s behavior not only judges the behavioral outcome but also judges the behavioral intentions and motivations of other individuals, thus affects the final individual behavioral decision. These studies also fully demonstrate that a single consideration of the impact of material gain on the node is not sufficient. The authors in Reference [35] pointed out that reciprocity can effectively encourage individuals to cooperate. Barta et al. [36] indicated that reciprocal altruistic can promote cooperation and nodes continue to help others after receiving help from others, which also occurs among large groups. An evolutionary game model among reciprocal participants and opportunistic participants was established in Reference [37]. It proves that reciprocal altruistic is the only evolutionary stability strategy when participants can observe the type of each other, regardless of whether the reciprocal participants can supervise the speculative behavior. A sequential reciprocal game model was proposed in Reference [38]. The model defines the cooperative willing function, analyzes whether the other party is a good faith behavior or a malicious behavior, determines the willingness to cooperate of nodes according to the goodwill of the other party and defines the utility function of the reciprocator. The utility function is divided into two parts: economic utility and reciprocal utility.

In the field of experimental economics, the existence of reciprocal altruistic was proved by experiments in Reference [39]. The participants who generate the game cooperative behavior are not rewarded by the reciprocator. The authors in Reference [40] studied a gift exchange game experiment. Some participants are found to have reciprocal behavior which can affect the final game outcome.

In the field of biology, the non-relative mutual help behavior among biological individuals was described by the prisoner’s dilemma game model in Reference [41], which is defined as reciprocal altruistic behavior. Based on the evolutionary strategy of prisoner game evolution, a mathematical model of altruistic reciprocal evolution was established in Reference [42] to explain the generation of reciprocal altruistic behaviors in biological nature.

It can be seen from the above that there are some researches on the reciprocal altruistic in many fields and the reciprocal altruistic affects individual behaviors. The authors introduced behavior economic to social networking and crowd sourcing in Reference [43,44] and achieved good results. But according to the author’s research, there is currently no literature on the use of reciprocal altruistic for VANET. Therefore, based on the reciprocal altruistic of behavioral economics, this paper proposes a new message transmission cooperation guarantee mechanism based on the behavioral economics [45]. The detailed description of the mechanism is given as follows.

## 3. System Modeling

The system model proposed in this paper is inspired by a biological experiment. In the field of biology, reciprocal altruistic was first observed in an experiment on vampire bats feeding back similar species [46]. In this paper, the experiment is mapped to the cooperation mechanism in VANETs and the reciprocal altruistic behavior of the node is explored. A new message transmission cooperation guarantee mechanism different from the traditional one is proposed.

### 3.1. The Introduction of Reciprocal Altruistic

Back-feeding behavior among vampire bats is often regarded as an example of reciprocal altruistic [47,48]. This paper uses an experiment on vampire bats [48] to illustrate the problem. Some experimental observations are listed as follows:

(A) In a group of eight bats, five bats are removed from the group before feeding and returned to the group at dawn. It is subsequently observed that the five bats which are not fed received feedback from the group;
(B) In another bat group, one bat is removed from the group every night before feeding and each bat is removed from the group at least twice. It is found that the bats which are fed back are more inclined to feed back to other bats in later experiments. And a male bat in the group who does not make a back-feeding behavior and is weakly connected to other bats did not receive feedback from other bats even if it needs to be feedback.

If the vampire bats in this experiment are completely rational, then they will not help other bats in the event, which may reduce their own survival opportunity (the bats that are fed are theoretically more likely to survive than the starving bats) and should not punish the bats that exhibit “malicious” behavior.

Based on this experiment, the paper proposes BMCGM. The mapping relationship is shown in Table 1.

### 3.2. System Model

**Figure 1:**
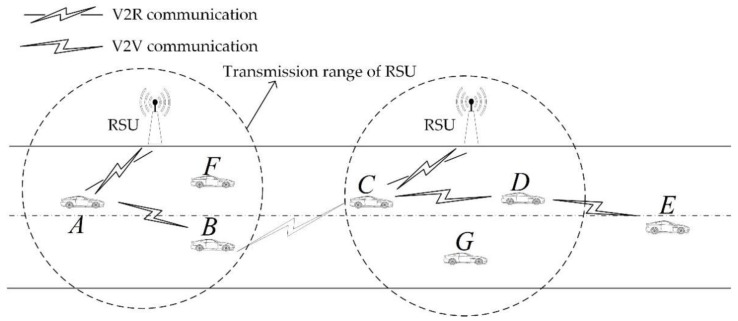
Network model.

As shown in Figure 1, our network model mainly includes vehicle nodes and RSU. The vehicle node can communicate directly with the RSU in the range (Vehicle to RoadSide Unit communication, V2R communication) and the vehicle node can also communicate with the vehicle node within the communication distance by the vehicle unit (Vehicle to Vehicle communication, V2V communication), or can communicate with other vehicle nodes which outside its communication range through other vehicle nodes. When the vehicle node communicates through one or more hops, a tree communication structure from the source node to the destination node is established and the vehicle node can perform message transmission along the established tree structure.

In our model, the RSUs store some information about the nodes’ interaction and an interaction message InterMsg={MsgNo,NID,SID,DID,Time,IsCoop,PK} is sent to the RSU whenever the node pair completes an interaction, where MsgNo is the serial number of the message, NID indicates the unique ID of the node that submit the message, SID indicates the unique ID of the message sending node, DID indicates the unique ID of the message receiving node,Time indicates the time that the interaction occurred and IsCoop indicates whether the receiving node cooperates in the interaction and PK denotes the private key of the node that submits the message to RSU, PK can be used to verify the correctness of the message. This interaction information is submitted in order to obtain the necessary information from the RSU when nodes are trying to form a communication link with other nodes later. To simplify the model, we assume that the node will honestly submit the interaction information. In order to clearly explain the contents of this section, the main parameters used in this section are shown in Table 2.

In this paper, BMCGM is divided into two phases: the cooperation formation phase and the multi-hop relay phase. The focus is to study how to promote the cooperative behavior of nodes under the altruistic reciprocity, so that the node can participate in multi-hop message transmission by the formed network graph. Thus, the phase of cooperation formation is the focus of this paper. Each phase is described as follows in conjunction with the Figure 1.

Cooperation formation phase: as shown in Figure 1, we assume that node *A* needs to send a message to node *E*. Since node E is not in the communication range of node *A*, it is necessary to construct a multi-hop communication link from node *A* to node *E*. In the communication range of node *A*, there are node *B* and node *E*. Node *A* acquires $\Pi_{BA}$ of node *B* and $\Pi_{EA}$ of node *E* through the adjacent RSU and simultaneously calculates the $\Gamma_{A}$ that may be obtained, then calculates $\Lambda_{A}$ that may be obtained by cooperation with node *B* and node *E* respectively according to $\Gamma_{A}$, $\Pi_{BA}$ and $\Pi_{EA}$. At last, node *A* determines which node to cooperate based on $\Lambda_{A}$. In Figure 1, node *A* finally selects node *B* as its next hop. Node *B* repeats this phase until destination node *E* is within its communication range.

Multi-hop relay phase: as shown in Figure 1, after the completion of the cooperation formation phase, the communication link from node *A* to node *E* is A→B→C→D→E, that is, node *A* sends a message to node *E* along the link.

## 4. The Details of BMCGM

This section is the core of the paper, including the node utility function design based on reciprocal altruistic, the analysis of the influence of the reciprocal altruistic of the node on its behavior and the design of NFG algorithm based on reciprocal altruistic. To clearly explain the contents of this section, the main parameters used are shown in Table 2.

### 4.1. Reciprocal Altruistic-Based Utility function

The main purpose of this section is to design a utility function that promotes node cooperation. For any vehicle node *A* in the network G(A,ε) and ε denotes the communication links among nodes. When it successfully participates in the cooperative transmission of the message, it will get a positive incentive value. When designing the utility function, traditional message cooperation mechanisms usually only consider the transmission effort of nodes and design economics utility functions accordingly. Because of the existence of reciprocal altruistic, this paper considers the transmission cost of nodes and the cooperation effort of nodes. Based on this, a utility function including economic utility and reciprocal utility is designed.

#### 4.1.1. Nodes’ Transmission Effort

To evaluate the transmission cost of the node, that is, the economic utility, we need to analyze the nodes in the message transmission link. It is assumed that the economic utility of each node participating in the message transmission is represented as $\Gamma_{i}$:

(1) $$\Gamma_{i}=\frac{\xi_{i}\cdot \tau_{i}}{\varphi_{i}}\;$$

where $\xi_{i}$ is the number of messages which node $A_{i}$ participates in the cooperative transfer, $\tau_{i}$ is the average transmission energy loss of the message of node $A_{i}$, $\varphi_{i}$ is the average message carrying time of node $A_{i}$. When a node consumes the more energy in order to participate in message transmission, the more effort it exerts. When the node carries the message for a long time without forwarding the message, the possibility that the message is transmitted to the destination node before the end of the TTL will decrease. So, the longer the average message carrying time of the node is, the less the transmission effort is paid.

#### 4.1.2. Nodes’ Reciprocal Cooperation

Reciprocal utility refers to the psychological utility of nodes due to reciprocal cooperation. In order to analyze the reciprocal utility of nodes for mutual cooperation, we first define the cooperative willingness $\Pi_{ij}$ of node $A_{i}$ to node $A_{j}$.

(2) $$\Pi_{ij}=\left\{ \begin{array}{l} \frac{\Omega_{{}_{ij}}^{C}}{\Omega_{{}_{ij}}^{C}+\Omega_{ij}^{D}+1}\;,\;\Omega_{{}_{ij}}^{C}\neq 0\;or\;\Omega_{ij}^{D}\neq 0\\ \;\frac{1}{2}\;,\;other \end{array}\right.\;$$

where $\Omega_{{}_{ij}}^{C}$ and $\Omega_{ij}^{D}$ respectively indicate the node cooperation and non-cooperation times interacting with the other nodes. It can be seen from Equation (2) that Π of the node depends on the cooperation and non-cooperation times of the node. The denominator part is added 1 to prevent Π value becomes 0 or 1 when the nodes have only one interaction. When there is no interaction between node pair, the default value is $\frac{1}{2}$.

At the same time, since the node has reciprocal altruistic properties, the following equation is established:

(3) $$\Pi_{ij}=\delta_{i}\cdot \Pi_{ji}\;$$

where δ,Π∈[0,1], $\delta_{i}$ is reciprocal altruistic factor, means the level of reciprocal altruistic of node $A_{i}$, when $\delta_{i}$ is larger, Π given to other nodes by node $A_{i}$ will increase accordingly. On the other hand, the smaller the $\delta_{i}$ is, the less Π of node $A_{i}$ will be reduced, that is, the node with higher degree of reciprocity, Π of $A_{i}$ given to other nodes is also higher.

According to Π of the node and the reciprocal altruistic, the reciprocal utility function of the node $A_{i}$ is given by:

(4) $$\Phi_{i}=\sum_{A_{j}\in G,j\neq i}\Pi_{ji}+\gamma \cdot \Gamma_{i}\cdot \sum_{A_{j}\in G,j\neq i}\Pi_{ji}\;$$

It can be seen from Equation (4) that the node’s psychological utility function is related to Π of other nodes in addition to its own $\Gamma_{i}$, which is because of nodes with reciprocal altruistic attributes, Π of other nodes will increase its degree of willingness to cooperate, the greater Π of other nodes is, the higher the willingness to cooperate is and the greater $\Phi_{i}$ is. Where γ is reciprocal altruistic cooperation influence coefficient, indicates the potential impact of reciprocal cooperation among nodes on their $\Phi_{i}$. It is because in order to complete the successful transmission of the message, it is often necessary to cooperate with each node on the link. The strategy *S* selected by each node may be related to the behavior of other nodes, which will have some potential impacts and γ∈[0,1), γ=0 means the $\Phi_{i}$ of nodes is only related to Π of other nodes.

#### 4.1.3. Utility Function

Based on $\Gamma_{i}$ and $\Phi_{i}$ of the node, we define the utility function of the node as follows:

(5) $$\begin{array}{c} \Lambda_{i}=\alpha \;\cdot \frac{\xi_{i}\cdot \tau_{i}}{\varphi_{i}}+\beta \;\cdot \sum_{A_{j}\in G,j\neq i}\Pi_{ji}\\ \;+\beta \cdot \gamma \cdot \frac{\xi_{i}\tau_{i}}{\varphi_{i}}\cdot \sum_{A_{j}\in G,j\neq i}\Pi_{ji}-C_{i} \end{array}$$

where α and β represent the node’s economic utility proportion coefficient and the reciprocal utility proportion coefficient in the total utility of the node, and:

(6) α+β=1 

where $C_{i}$ represents the cost of node $A_{i}$, and:

(7) $$C_{i}=\frac{1}{2}\mu \cdot ({\left(\Gamma_{i}\right)}^{2}+\Pi_{i}{}^{2})\;$$

When α=1, β=0, it indicates that the node is a node that is assumed by traditional economics to maximize its own $\Phi_{i}$ and in the cost $C_{i}$, $\Pi_{i}=0$. This is because the nodes assumed in traditional economics do not have reciprocal cooperative behavior.μ indicates the cost coefficient, when μ gets larger, the cost of cooperation among nodes is larger.

According to the Equation (5), the cost of node $A_{i}$ is related to its $\Phi_{i}$ and cooperative efforts brought about by reciprocal cooperation.

**Figure 2:**
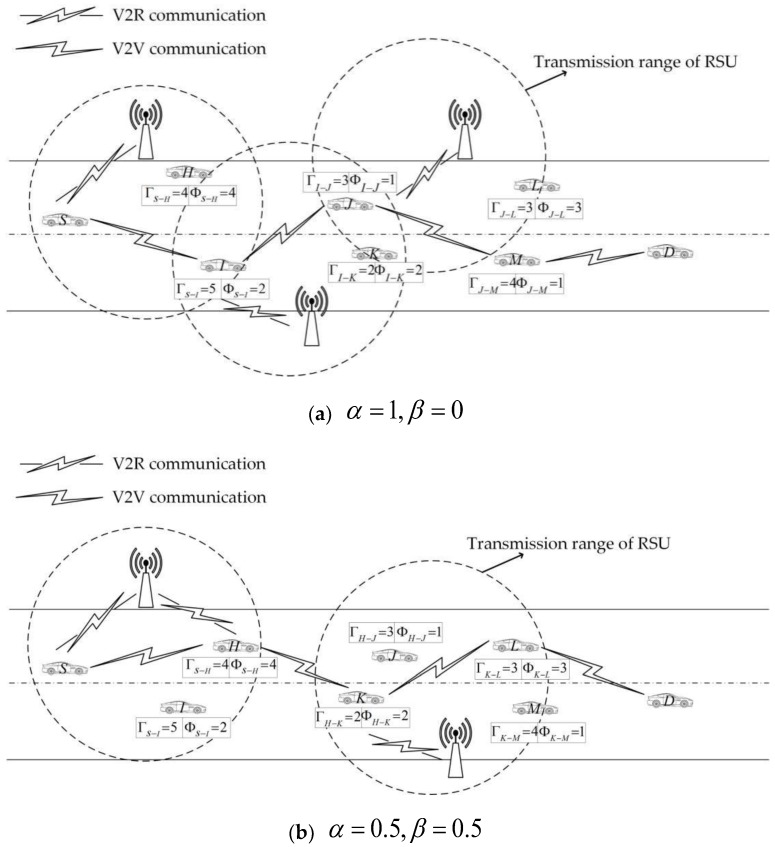
Selection of relay nodes.

We use Figure 2 to illustrate the effect of Φ on the node’s behavior. Suppose node S needs to send a message to node D:

When α=1, β=0, c=0.2, nodes are assumed in traditional economics to maximize Λ.

For node S:

If S chooses to cooperate with node I, the possible utility of node S is:

$\Lambda_{S-I}=1\times 5+0\times 2-0.5\times 0.2\times 5^{2}=2.5$;

If S chooses to cooperate with node H, the possible utility of node S is:

$\Lambda_{S-H}=1\times 4+0\times 4-0.5\times 0.2\times 4^{2}=2.4$;

Since $\Lambda_{S-I}>\Lambda_{S-H}$, node S may choose to cooperate with node I.

Assuming node I cooperates, then for node I:

If I chooses to cooperate with node J, the possible utility of node I is:

$\Lambda_{I-J}=1\times 3+0\times 1-0.5\times 0.2\times 3^{2}=2.1$;

If I chooses to cooperate with node K, the possible utility of node I is:

$\Lambda_{I-K}=1\times 2+0\times 2-0.5\times 0.2\times 2^{2}=1.6$;

Since $\Lambda_{I-J}>\Lambda_{I-K}$, node I may choose to cooperate with node J.

Assuming node J cooperates, then for node J:

If J chooses to cooperate with node M, the possible utility of node J is:

$\Lambda_{J-M}=1\times 4+0\times 1-0.5\times 0.2\times 4^{2}=2.4$;

If J chooses to cooperate with node L, the possible utility of J is:

$\Lambda_{J-L}=1\times 3+0\times 3-0.5\times 0.2\times 3^{2}=2.1$;

Since $\Lambda_{J-M}>\Lambda_{J-L}$, node J may choose to cooperate with node M.

Since node D is located at the transmission range of node M, node M no longer performs cooperative node selection and transmits the message directly to node D. The resulting communication link at this point may be: S→I→J→M→D;

When α=β=0.5,c=0.2, the nodes are with reciprocal altruistic.

For node S:

If S chooses to cooperate with node I, the possible utility of node S is:

$\Lambda_{S-I}=0.5\times 5+0.5\times 2-0.1\times (5^{2}+2^{2})=0.6$;

If S chooses to cooperate with node H, the possible utility of node S is:

$\Lambda_{S-H}=0.5\times 4+0.5\times 4-0.1\times (4^{2}+4^{2})=0.8$

Since $\Lambda_{S-I}<\Lambda_{S-H}$, node S may choose to cooperate with node H.

Assuming node H cooperates, then for node H:

If H chooses to cooperate with node J, the possible utility of node H is:

$\Lambda_{H-J}=0.5\times 3+0.5\times 1-0.1\times (3^{2}+1^{2})=1$;

If H chooses to cooperate with node K, the possible utility of node H is:

$\Lambda_{H-K}=0.5\times 2+0.5\times 2-0.1\times (2^{2}+2^{2})=1.2$;

Since $\Lambda_{H-J}<\Lambda_{H-K}$, node H may choose to cooperate with node K.

Assuming node K cooperates, then for node K:

If K chooses to cooperate with node M, the possible utility of node K is:

$\Lambda_{K-M}=0.5\times 4+0.5\times 1-0.1\times (4^{2}+1^{2})=0.8$;

If K chooses to cooperate with node L, the possible utility of node K is:

$\Lambda_{K-L}=0.5\times 3+0.5\times 3-0.1\times (3^{2}+3^{2})=1.2$;

Since $\Lambda_{K-M}<\Lambda_{K-L}$, node K may choose to cooperate with node L.

Since node D is located at the transmission range of node L, node L no longer performs cooperative node selection and transmits the message directly to node D. The resulting communication link at this point may be: S→H→K→L→D;

### 4.2. Reciprocal Altruistic-Based NFG Algorithm

The main purpose of this section is to design NFG algorithm, which simulates node interactions considering reciprocal altruistic of nodes.

Since the vehicle nodes in the network are determined independently of each other, they can decide whether to use a node as their next hop, that is, whether to establish a communication link with a certain node. We first define the strategy set of the vehicle node $A_{i}$, denoted by $\Delta_{i}=\{s_{1},s_{2},\ldots s_{n}\}$. The set $\Delta_{i}$ contains all the strategy possibilities of node $A_{i}$ in the current network environment and node $A_{i}$ can select a strategy s from the set $\Delta_{i}$ to change the currently executed strategy. Assume that the strategy currently used by node $A_{i}$ is $s_{i}\in \Delta_{i}$, the network topology at this time is represented as $G_{s_{i}^{c},-s_{{}^{i}}^{c}}$, where $-s_{i}$ indicates strategy that other nodes are taken at this time. When node $A_{i}$ selects a new strategy $s_{j}\in \Delta_{i}$ and other nodes keep the current strategy unchanged, the new network strategy can be represented as $G_{new}=G_{s_{i}^{c},-s_{{}^{i}}^{c}}+s_{ij}$.

When node $A_{i}$ selects strategy $s_{j}=(A_{i},A_{j})\in \Delta_{i}$, that is, node $A_{i}$ attempts to establish a link with node $A_{j}$. In the traditional economics persecution, if node $A_{j}$ agrees to establish a link with node $A_{i}$, its utility will be reduced, node $A_{j}$ may refuse to establish link with node $A_{i}$. However, under the concept of behavioral economics, because of the existence of the reciprocal altruistic, even if it may reduce the utility of node $A_{j}$, node $A_{j}$ will still choose to cooperate. We define the reciprocal cooperation strategy as follows:

**Definition** **1.** *Reciprocal cooperation strategy*$s_{ij}$*. When strategy*$s_{ij}=(A_{i},A_{j})\in \Delta_{i}$*makes*$\Gamma_{i}(G_{s_{i}^{c},-s_{{}^{i}}^{c}}+s_{ij})<\Gamma_{i}(G_{s_{i}^{c},-s_{{}^{i}}^{c}})$*and because of*$\Phi_{i}$*which makesthe total utility*$\Lambda_{i}(G_{s_{i}^{c},-s_{{}^{i}}^{c}}+s_{ij})\geq \Lambda_{A_{j}}(G_{s_{i}^{c},-s_{{}^{i}}^{c}})$*, the strategy*$s_{ij}$*is a reciprocal cooperation strategy, the set of the strategy*$s_{ij}$*is represented by the set*$\Delta_{i}^{}{}^{*}$and$\Delta_{i}^{}{}^{*}\subseteq \Delta_{i}$.

The reciprocal cooperation strategy $s_{ij}$ causes node $A_{i}$ to request the link with node $A_{j}$ because the strategy ultimately increases the utility of node $A_{i}$. Based on the reciprocal cooperation strategy, we define the optimal reciprocal cooperation strategy set as follows:

**Definition** **2.** *Optimal reciprocal cooperation strategy*$s_{ij}^{*}$*. When the reciprocal cooperation strategy*$s_{ij}^{*}\subseteq \Delta_{i}^{}{}^{*}$makes the utility value$\Lambda_{i}(G_{s_{i}^{c},-s_{{}^{i}}^{c}}+s_{ij}^{*})\geq \Lambda_{i}(G_{s_{i}^{c},-s_{{}^{i}}^{c}})$*of the node*$A_{i}$*,*$s_{ij}^{*}\subseteq \Delta_{i}^{}{}^{*}$, the strategy$s_{ij}^{*}$*is an optimal reciprocal cooperation strategy and the set of the strategy*$s_{ij}^{*}$*is represented by the set*${\hat{\Delta }}_{i}{}^{*}$,${\hat{\Delta }}_{i}{}^{*}\subseteq \Delta_{i}^{}{}^{*}$.

#### 4.2.1. The Influence of δ and β

In the process of NFG, due to the existence of δ of the node, the strategy selection of the node will be affected by the reciprocal altruistic. When δ changes, the optimal reciprocal cooperation strategy set ${\hat{\Delta }}_{i}{}^{*}\subseteq \Delta_{i}^{}{}^{*}$ of the node may also change accordingly. So that the final optimal reciprocal cooperation strategy can make each vehicle node to a select the most suitable one (if the strategy is feasible) according to the current network conditions and increase the utility of the node itself. Therefore, our NFG algorithm is an algorithm based on the optimal reciprocal cooperation strategy ${\hat{\Delta }}_{i}{}^{*}$ of nodes and the optimal reciprocal cooperation strategy set ${\hat{\Delta }}_{i}{}^{*}$ is related to δ. Next, we analyze the impact of δ on node cooperative behavior and its utility.

A. The influence of δ on nodes’ cooperation behavior

Since the cooperative behavior of the node is related to Π of the node, in order to analyze the influence of δ on the node’s cooperative behavior, we substitute Equations (3) and (4) into Equation (5). We find partial derivatives for Γ and Π respectively and obtain possible extreme points $\Gamma_{i}{}^{*}$ and $\Pi_{i}{}^{*}$, which we will further analyze in later sections:

(8) $$\Gamma_{i}{}^{*}=\frac{\alpha \cdot \mu \cdot \delta_{i}^{2}+\beta^{2}\cdot \gamma }{\delta_{i}^{2}\cdot \mu^{2}-\beta^{2}\cdot \gamma^{2}}\;$$

(9) $$\Pi_{i}{}^{*}=\frac{\beta \cdot \delta_{i}\cdot (\mu +\alpha \gamma )}{\delta_{i}^{2}\cdot \mu^{2}-\beta^{2}\cdot \gamma^{2}}\;$$

When building NFG algorithm, a very important part is needed to analyze when the node chooses a reciprocal cooperation strategy. It can be known from Equation (9) that Π of the node will be larger than 0 only when certain conditions are met. That is, the reciprocal cooperative behavior of the node needs to meet certain conditions. We prove it by Theorem 1.

**Theorem** **1.** *When* $\delta_{i}$ *of the node* $A_{i}$ *is* $\delta_{i}>\frac{\beta \cdot \gamma }{\mu }$ *,* Π *of the node* $\Pi_{i}{}^{*}>0$ *, the node* $A_{i}$ *will cooperate.*

**Proof.**  Known by Equation (9), when $\delta_{i}>\frac{\beta \cdot \gamma }{\mu }$,there are:

$\beta \cdot \delta_{i}\cdot (\mu +\alpha \gamma )>0$, $\delta_{i}^{2}\cdot \mu^{2}-\beta^{2}\cdot \gamma^{2}>0$,

∴$\Pi_{i}{}^{*}>0$; When $\delta_{i}<\frac{\beta \cdot \gamma }{\mu }$, there are: $\beta \cdot \delta_{i}(\mu +\alpha \cdot \gamma )>0$, where $\delta_{i}^{2}\cdot \mu^{2}-\beta^{2}\cdot \gamma^{2}<0$,

∴$\Pi_{i}{}^{*}<0$; that is, when $\delta_{i}$ of the node $A_{i}$ is $\delta_{i}>\frac{\beta \cdot \gamma }{\mu }$, Π of the node $\Pi_{i}{}^{*}>0$, the node $A_{i}$ will cooperate. □

From Theorem 1, it can be known that the reciprocal cooperation behavior of the node will only occur when its level of reciprocal altruistic $\delta_{i}>\frac{\beta \cdot \gamma }{\mu }$. Since γ and μ are relatively stable constants, the cooperative behavior of the node is mainly related to $\delta_{i}$ and β. Reasonably designing β based on $\delta_{i}$, the node’s cooperation behavior can be promoted.

B. The influence of δ on nodes’ utility

It can be seen from Equations (3) and (5) that the utility $\Lambda_{i}$ of the node is related to the reciprocal altruistic $\delta_{i}$ of the node. When the nodes choose to cooperate, the reciprocal cooperation strategy set $\Delta_{i}^{}{}^{*}$ at this time may contain multiple strategies and the node will select an optimal reciprocal cooperation strategy $s_{j}^{*}$ to maximize its total utility $\Lambda_{i}$. We prove that $\Lambda_{i}$ is related to $\delta_{i}$ of the node by Theorem 2.

**Theorem** **2.** $\Gamma_{i}{}^{*}$ *and* $\Pi_{i}{}^{*}$ *obtained by Equation (8) and (9) are the optimal economic utility and the optimal cooperative willingness and* $\Lambda_{i}$ *of the node is the largest at this time.*

**Proof.**  According to Equation (5), find the partial derivative of Γ and Π, there be:

(10) $$\frac{\partial \Lambda_{i}}{\partial \Gamma_{i}}=\alpha +\frac{\beta \cdot \gamma \;\cdot \Pi_{i}}{\delta_{i}}-\mu \cdot \Gamma_{i}\;$$

(11) $$\frac{\partial \Lambda_{i}}{\partial \Pi_{i}}=\frac{\beta \cdot (1+\gamma \Gamma_{i})}{\delta_{i}}-\mu \cdot \Pi_{i}\;$$

let $\frac{\partial \Lambda_{i}}{\partial \Gamma_{i}}$, $\frac{\partial \Lambda_{i}}{\partial \Pi_{i}}=0$, then:

$$\Gamma_{i}{}^{*}=\frac{\alpha \cdot \mu \cdot \delta_{i}^{2}+\beta^{2}\cdot \gamma }{\delta_{i}^{2}\cdot \mu^{2}-\beta^{2}\cdot \gamma^{2}},\;\Pi_{i}{}^{*}=\frac{\beta \cdot \delta_{i}(\mu +\alpha \cdot \gamma )}{\delta_{i}^{2}\cdot \mu^{2}-\beta^{2}\cdot \gamma^{2}},$$

also ∵ $\frac{\partial^{2}\Lambda_{i}}{\partial \Gamma_{i}{}^{2}}=\;-\mu$, $\frac{\partial^{2}\Lambda_{i}}{\partial \Gamma_{i}\partial \Pi_{i}}=\;\frac{\beta \cdot \gamma \;}{\delta_{i}}$, $\frac{\partial^{2}\Lambda_{i}}{\partial \Pi_{i}{}^{2}}=-\mu$,

$∴\frac{\partial^{2}\Lambda_{i}}{\partial \Gamma_{i}{}^{2}}\cdot \frac{\partial^{2}\Lambda_{i}}{\partial \Pi_{i}{}^{2}}-{\left(\frac{\partial^{2}\Lambda_{i}}{\partial \Gamma_{i}\partial \Pi_{i}}\right)}^{2}=\mu^{2}-\;\frac{\beta^{2}\cdot \gamma^{2}\;}{\delta_{i}{}^{2}}$,

also ∵ According to Theorem 1, when node $A_{i}$ chooses to cooperate, there is $\delta_{i}>\frac{\beta \cdot \gamma }{\mu }$, then

$$\frac{\partial^{2}\Lambda_{i}}{\partial \Gamma_{i}{}^{2}}\cdot \frac{\partial^{2}\Lambda_{i}}{\partial \Pi_{i}{}^{2}}-{\left(\frac{\partial^{2}\Lambda_{i}}{\partial \Gamma_{i}\partial \Pi_{i}}\right)}^{2}>0,$$

also ∵ $\frac{\partial^{2}\Lambda_{i}}{\partial \Gamma_{i}{}^{2}}=\;-\mu <0$, ∴ According to the LaGrange multiplier method, it can be concluded that: $\Gamma_{i}{}^{*}$ and $\Pi_{i}{}^{*}$ are the optimal economics utility and the optimal cooperative willingness and $\Lambda_{i}$ of the node is the largest at this time. □

Theorem 2 proves that the nodes with reciprocal altruistic attributes always have optimal $\Gamma_{i}{}^{*}$ and optimal $\Pi_{i}{}^{*}$ when they cooperate, which makes their $\Lambda_{i}$ maximal. In the reciprocal altruistic-based NFG proposed in this paper, the node needs to choose the appropriate optimal reciprocal cooperation strategy to maximize its total utility. Therefore, we need to analyze the conditions under which the node can obtain optimal $\Gamma_{i}{}^{*}$ and optimal $\Pi_{i}{}^{*}$. We prove it by Theorem 3.

**Theorem** **3.** *When* $\delta_{i}\to {\frac{\beta \cdot \gamma }{\mu }}^{+}$ *, node’s optimal* $\Gamma_{i}{}^{*}$ *and* $\Pi_{i}{}^{*}$ *are the largest and utility* $\Lambda_{i}$ *is largest at this time.*

**Proof.**  When $\delta_{i}\to {\frac{\beta \cdot \gamma }{\mu }}^{+}$,there be:

$\underset{\delta_{i}\to \frac{2\beta \cdot \gamma }{\mu }}{\lim}\delta_{i}^{2}\cdot \mu^{2}-\beta^{2}\cdot \gamma^{2}=0,$$\underset{\delta_{i}\to \frac{\beta \cdot \gamma }{\mu }}{\lim}\alpha \cdot \mu \cdot \delta_{i}^{2}+\beta^{2}\cdot \gamma =c_{1},$$\underset{\delta_{i}\to \frac{\beta \gamma }{\mu }}{\lim}\beta \delta_{i}(\mu +\alpha \gamma )=c_{2},$ and $c_{1},c_{2}\in Ν$.

∴$\underset{\delta_{i}\to \frac{2\beta \gamma }{\mu }}{\lim}\Gamma_{i}{}^{*}=\infty$, $\underset{\delta_{i}\to \frac{2\beta \gamma }{\mu }}{\lim}\Pi_{i}{}^{*}=\infty$, ∴ node’s optimal $\Gamma_{i}{}^{*}$ and $\Pi_{i}{}^{*}$ are the largest and utility $\Lambda_{i}$ is largest at this time. □

It can be seen from Theorems 1–3 that when $\delta_{i}\to {\frac{\beta \cdot \gamma }{\mu }}^{+}$, optimal $\Gamma_{i}{}^{*}$ of the node and optimal $\Pi_{i}{}^{*}$ are the largest. In this way, we can get an inspiration that β should change with the change of $\delta_{i}$ of the node, in the case that the cooperation influence coefficient γ and μ are constant. The node can obtain the optimal $\Gamma_{i}{}^{*}$ and the optimal $\Pi_{i}{}^{*}$.

C. The influence of β on node’s cooperation behavior

It can be seen from the above analysis that when the level of altruistic property $\delta_{i}$ of the node is certain, under the condition that $\delta_{i}>\frac{\beta \cdot \gamma }{\mu }$ is satisfied (that is, under the condition that the node will perform the reciprocal cooperation behavior), there is $\Pi_{i}{}^{*}\propto \beta$, that is, the increment of β can promote the node to choose the reciprocal cooperation behavior, which we prove by Theorem 4.

**Theorem** **4.** *When* $\delta_{i}$ *of the node* $A_{i}$ *is constant, there is* $\Pi_{i}{}^{*}\propto \beta$ *. At this time, a single increase in* β *will promote the reciprocal cooperation behavior of the node.*

**Proof.**  According to Equation (9), there be:

$\frac{\partial \Pi_{i}{}^{*}}{\partial \beta }=\frac{2\beta \cdot \delta_{i}(\mu +\alpha \cdot \gamma )}{{\left(\delta_{i}^{2}\cdot \mu^{2}-\beta^{2}\cdot \gamma^{2}\right)}^{2}}$,

also ∵ ${\left(\delta_{i}^{2}\cdot \mu^{2}-\beta^{2}\cdot \gamma^{2}\right)}^{2}>0$, $2\beta \cdot \delta_{i}(\mu +\alpha \cdot \gamma )>0$,

∴$\frac{\partial \Pi_{i}{}^{*}}{\partial \beta }>0$, that is: $\Pi_{i}{}^{*}\propto \beta$. □

At this time, a single increase in β will promote the reciprocal cooperation behavior of the node.

Theorem 4 further demonstrates that while $\delta_{i}$ of the node is included in the message cooperative communication mechanism, β in the utility of the node also needs to be considered. Appropriate increment of β can improve the node’s optimal $\Gamma_{i}{}^{*}$ and optimal $\Pi_{i}{}^{*}$, thus promoting the reciprocal cooperation behavior of the nodes. We calculate the value of β by Algorithm 1.

Based on Algorithm 1, we can calculate the appropriate value of β nodes that with different level of reciprocal altruistic.

**Algorithm 1:** Computing_β**Initialize:** The level of reciprocal altruistic: δ, reciprocal utility proportion coefficient: β, cooperating influence coefficient: γ, Cost coefficient: μ1:  If $\delta \leq \frac{\beta \cdot \gamma }{\mu }$ then2:    β←0
3:  Else4:     While $\delta >\frac{\beta \cdot \gamma }{\mu }$ do5:       β←β + 0.01
6:     End7:  End8:  Return β

#### 4.2.2. Network Information Algorithm

Based on the above analysis of the reciprocal cooperation behavior of nodes, in the Behavior Economics-based NFG Algorithm proposed in this paper, each node will select the appropriate node to carry out the reciprocal cooperation behavior and construct the link from the source node to the destination node. Once the network topology G is determined, the message transmission process begins. The algorithm proposed in this paper is summarized as shown in Algorithm 2.

**Algorithm 2:** Reciprocal altruistic-based network formation algorithm**Inputs:** current node: CA; neighbor nodes set: N; total nodes set: A; The set of CA’s reciprocal cooperation strategy: $S_{CA}$; CA’s reciprocal cooperation strategy: $s_{CA}=\{CA,A_{x}\},s_{CA}\in S,A_{x}\in A$; CA’s utility:$\Lambda_{CA}$ **Output:** network graph:G
**Initialize state**
Source node $A_{i}$ wants to transmit packets to destination node $A_{j}$ through a set of nodes A
Reciprocal altruistic-based network formation game1:  $CA\leftarrow A_{i}$
2:  $G.add(A_{i})$
3:  While $N.getNode(A_{j})=\;=false$ do4:    if $S_{CA}\notin \emptyset$ then5:      if $\Lambda_{CA}(G+s_{CA})\geq \Lambda_{{}_{CA}}(G)$, $\forall s_{CA}\in S_{CA}$ then6:        $G.add(A_{x})$
7:        $CA\leftarrow A_{x}$
8:        N←CA.getNeighborNodes()
9:      End if10:    End if11:  End While 12:  Return G
**Cooperative packets transmission** Source node $A_{i}$ transmits packets to destination node $A_{j}$ through the formed link G Each node in link G gain a utility Λ based on the utility function mentioned before

## 5. Performance Evaluation

The experimental part of this paper uses the Free Mobility Model proposed in Reference [49] to construct a highway movement model scenario. In the experimental scenario, the expressway consists of two lanes with a length of 2 km and a width of 10 m. The values of parameters such as number of nodes, minimum speed and transmission range of nodes are diverse but for the fairness of the experiment, we use the same values as the comparison paper used in its experiment. At the initial moment, 23 vehicle nodes are randomly distributed at any position on the road, with a communication range of 300 m and moving from left to right. The minimum moving speed of the vehicle node is 10 m/s and the maximum moving speed is 60 m/s. When the vehicle node reaches the leftmost or rightmost end, the moving direction is changed, the experimental running time is 20 min and all the experimental data are running 1000 times. Take the average to eliminate the effects of some uncertainties. Specific experimental parameters are detailed in Table 3.

### 5.1. Cost Coefficient μ

**Figure 3:**
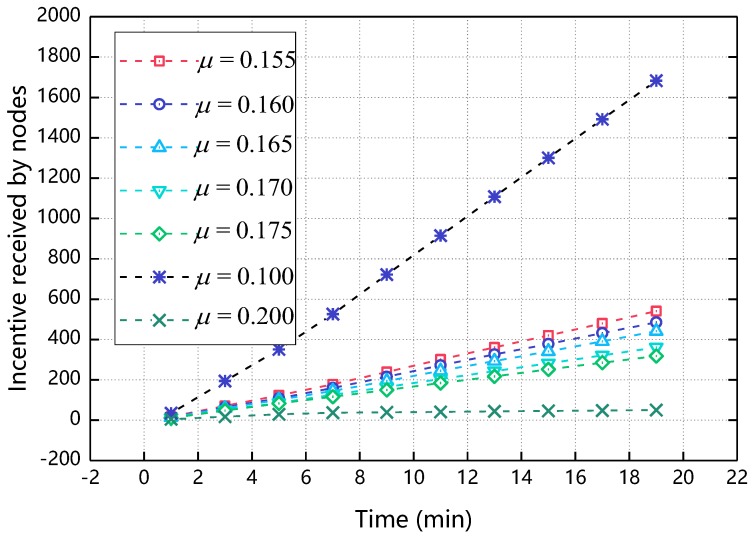
The impact of μ’s range on incentive received by nodes.

Cost coefficient affects the final utility of the node, indicating the degree of cost paid by the node due to cooperation. The smaller the value, the smaller the cost of cooperation among the nodes is. Since the size of the cost coefficient directly affects the incentive of the node and the incentive received ratio can reflect the proportion of participating nodes to a certain extent, this section discusses its influence from two aspects: incentive received by nodes and incentive received ratio. Before we discuss the value of μ in detail, let’s discuss the appropriate range of *µ*. As shown in Figure 3, when μ gradually increases from 0.1 to 0.2, the incentive received by nodes decreases rapidly. When μ = 0.1, the incentive received by nodes reaches about 1700 and when μ = 0.2, the incentive received by nodes is close to 0, incentive received by nodes becomes too large or too small will reduce the enthusiasm of node participation, thus we mainly discuss the situation when μ takes 0.155~0.175.

#### 5.1.1. The Impact of μ on Incentive Received by Nodes

**Figure 4:**
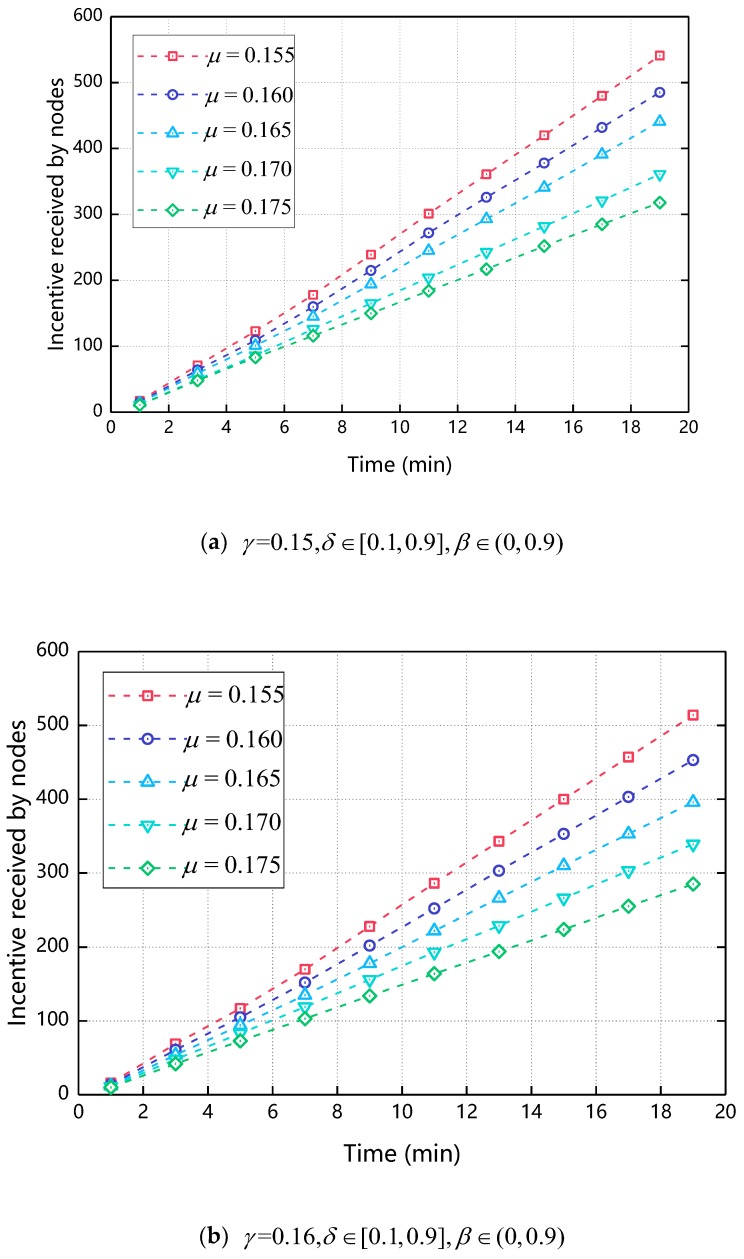

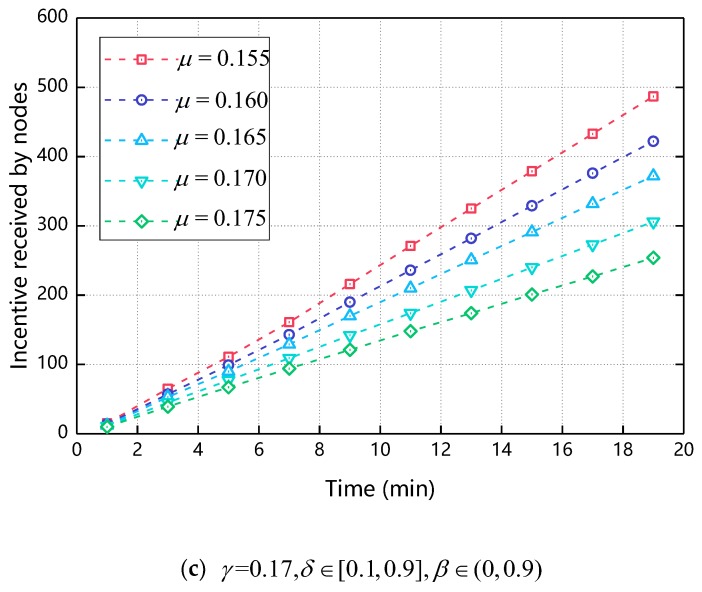
The impact of μ on incentive received by nodes.

When μ changes, the figure of the incentive received by nodes changes as shown in Figure 4. As can be seen from Figure 4. As the running time increases, the incentive obtained by the node also increases. When μ increasing from 0.155 to 0.175, the incentive finally obtained by the node decreases with the increment of μ, because when μ increases, the cost of cooperation of the node increases correspondingly, so that the ultimate incentive of the node is reduced. It also can be seen from Figure 3. When μ = 0.175, the final utility of the node will be too large; when μ = 0.155, the final utility of the node will be too small, so μ should be between 0.155 and 0.175. The specific value of μ is analyzed by the influence of μ on the received receive ratio of the node in the next section.

#### 5.1.2. The Impact of μ on Incentive Received Ratio

**Figure 5:**
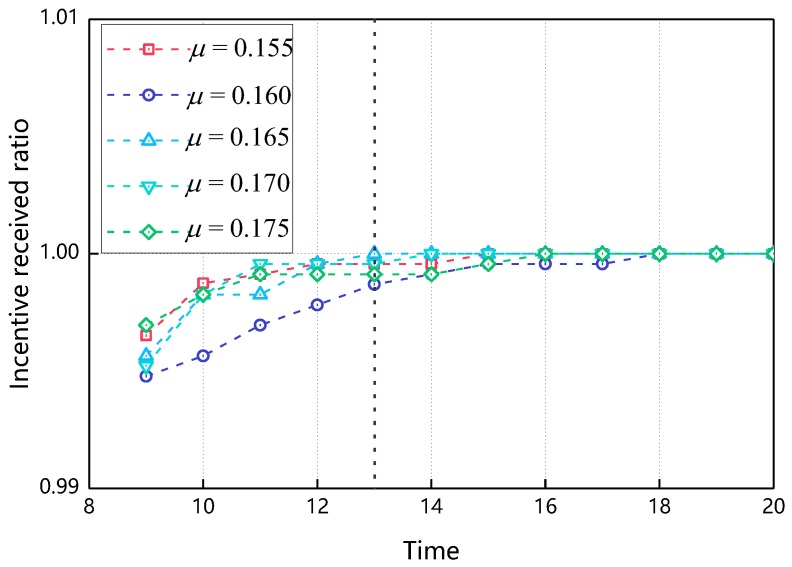
The impact of μ on incentive received ratio (γ=0.15,δ∈[0.1,0.9],β∈(0,0.9)).

Figure 5 shows the relationship of the received ratio over time as the μ changes. Incentive received ratio refers to the proportion of nodes that receive incentives in all nodes. Since the nodes only get the incentive after the cooperative behavior occurs, the incentive received ratio can reflect the proportion of node cooperation to a certain extent. As can be seen from Figure 5, as the simulation time increases, the incentive received ratio also increases and finally reaches 1, that is, all nodes participate in the cooperative transmission of the message. At the same time, it can be seen from Figure 5. At the 13th min, when μ = 0.165, the value of the incentive received ratio reaches 1 first, so in the subsequent experiments in this paper, 0.165 is taken.

**Figure 6:**
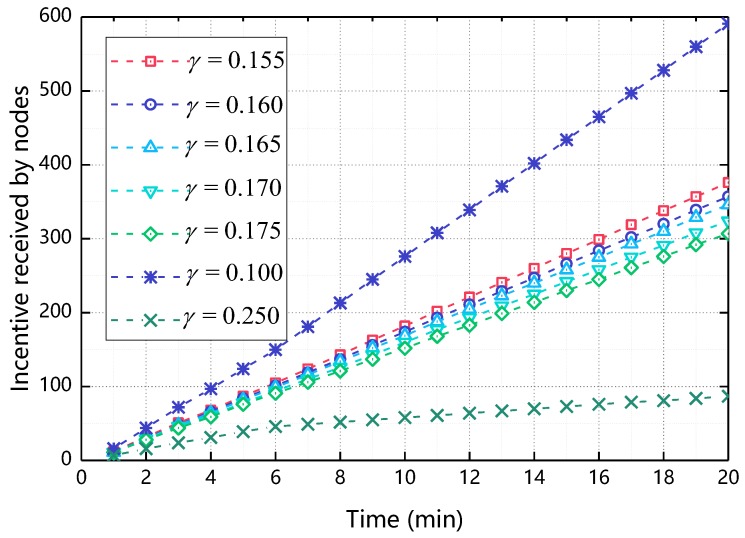
The impact of γ’s range on incentive received by nodes.

Before we discuss the value of γ in detail, let’s discuss the appropriate range of γ. As shown in Figure 6, when gradually increases from 0.1 to 0.25, the incentive received by nodes decreases rapidly. When γ = 0.1, the incentive received by nodes reaches about 600 and when γ = 0.25, the incentive received by nodes is less than 100, incentive received by nodes becomes too large or too small will reduce the enthusiasm of node participation, thus we mainly discuss the situation when γ takes 0.155~0.175.

### 5.2. Reciprocal Altruistic Cooperation Influence Coefficient γ

Cooperating influence coefficient γ is a coefficient that indicates the degree of influence of reciprocal cooperation among nodes on its Φ. The larger the value, the greater the influence of the reciprocal cooperation behavior among nodes. This section also analyzes the reasonable values from the two aspects: incentive received by nodes and incentive received ratio.

#### 5.2.1. The Impact of γ on Incentive Received by Nodes

**Figure 7:**
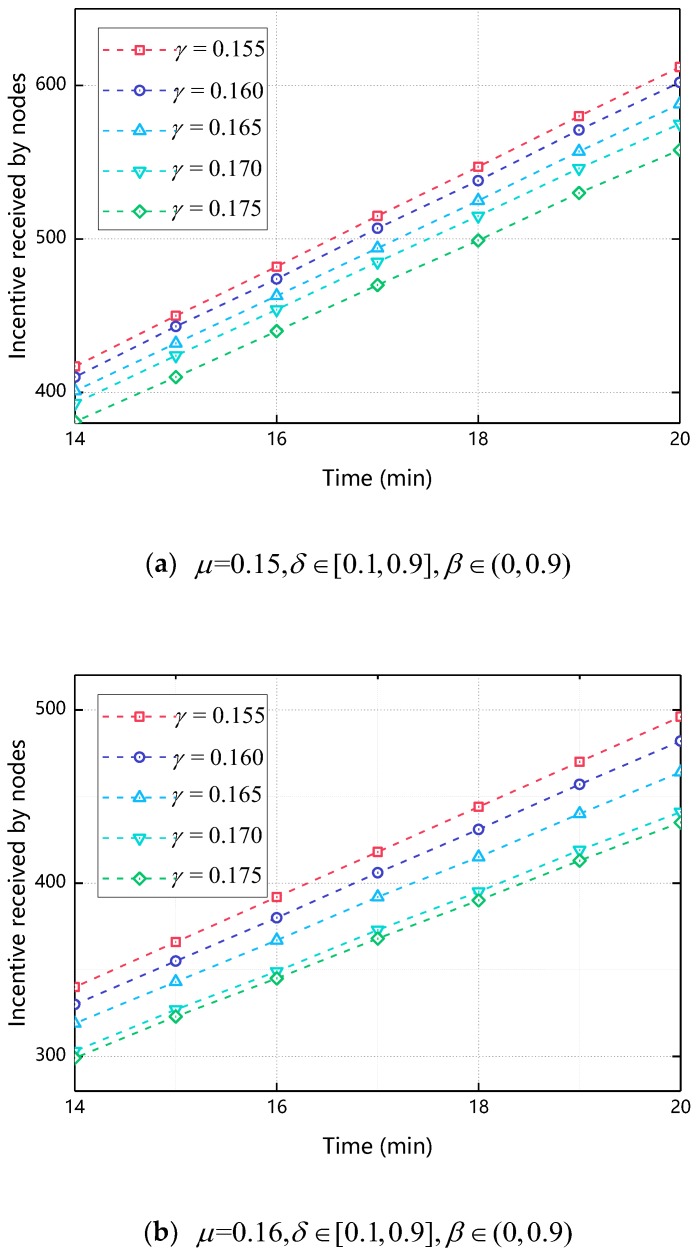

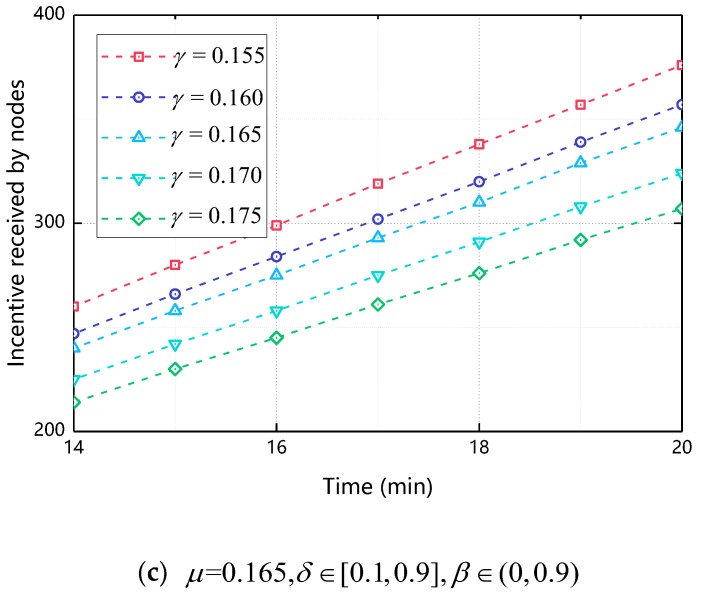
The impact of γ on incentive received by nodes.

Figure 7 shows the relationship between the incentive received by nodes and the time when γ changes. As shown in Figure 7, when γ increases, the final incentive value of the node decreases. This is because when γ increases, the greater the impact of cooperative behavior on Φ, the resulting reciprocal cooperation costs will increase accordingly, making the ultimate node’s incentive reduced. Compared with Figure 4, as γ increases, the final incentive change of the node is less. This is because γ only effects Φ of the node and the resulting reciprocal cooperation cost is less than the total cost of cooperation, so the degree of change is relatively small compared to Figure 4 We determine the range of values of γ is [0.165, 0.175] by Figure 6. The specific values of γ are discussed in the next section.

#### 5.2.2. The Impact of γ on Incentive Received Ratio

**Figure 8:**
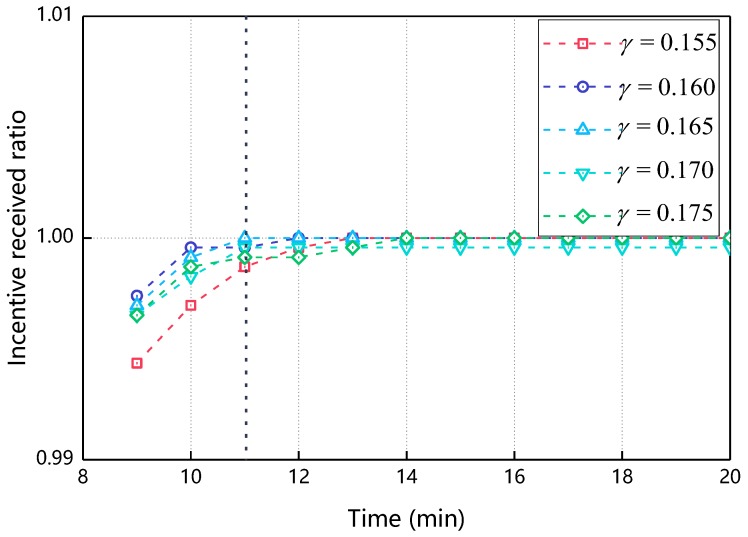
The impact of γ on incentive received ratio (μ=0.165,δ∈[0.1,0.9],β∈(0,0.9)).

Figure 8 shows the relationship of the incentive received ratio over time as γ changes. As can be seen from Figure 8, the incentive received ratio increases with time and finally reaches 1, that is, all nodes participate in cooperative message transmission. At the 11min, when γ = 0.165, the value of its incentive received ratio reaches 1 first, so in the subsequent experiments in this paper, γ takes 0.165.

### 5.3. Reciprocal Utility Proportion Coefficient β

β is a coefficient indicating the proportion of the node’s Φ in the total utility of the node. The larger the value is, the greater the proportion of the node’s Φ in its total utility is. We designed experiments based on Theorems 1 and 4 and explored reasonable values.

**Figure 9:**
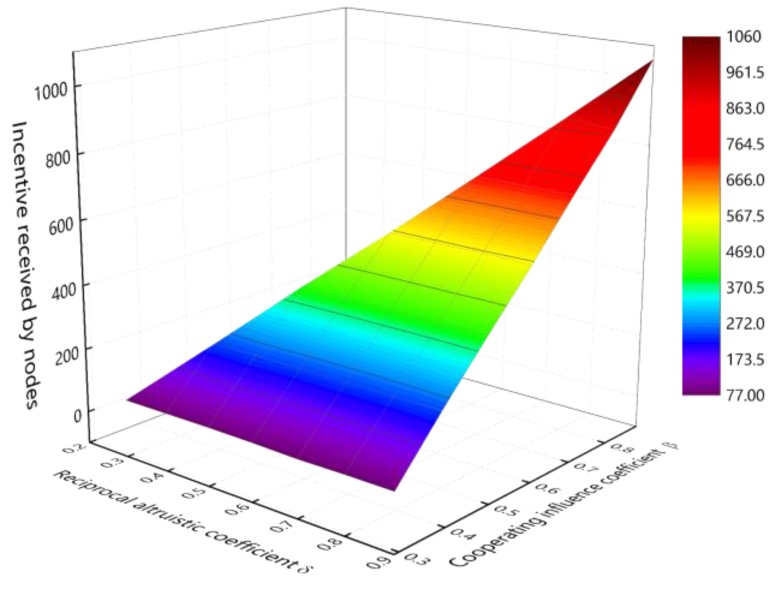
The impact of β on incentive received ratio (μ=γ=0.165,δ∈[0.1,0.9],β∈(0,0.9)).

As shown in Figure 9, the Z-axis represents the incentive received by nodes after running for 20 min. The X-axis represents the reciprocal altruistic coefficient δ of the node and the Y-axis represents β. Figure 9 shows the relationship between the incentive received by nodes and β. As can be seen from the figure, when δ = 0.9 and β→0.9, the total utility of the node is the largest, the same can be seen that, when δ = 0.5 or 0.7, β→0.5 or β→0.7, the total utility of the node is the largest. So, in the experiments that follow, we assume that β=δ−0.01.

### 5.4. BMCGM Compared with NGOMA

This section compares BMCGM with the traditional message cooperative communication mechanism NGOMA and analyzes the performance of BMCGM from four aspects: incentive received by nodes, incentive received ratio, delay ratio and transmitted packet ratio.

#### 5.4.1. Incentive Received by Nodes with Respect to Time

**Figure 10:**
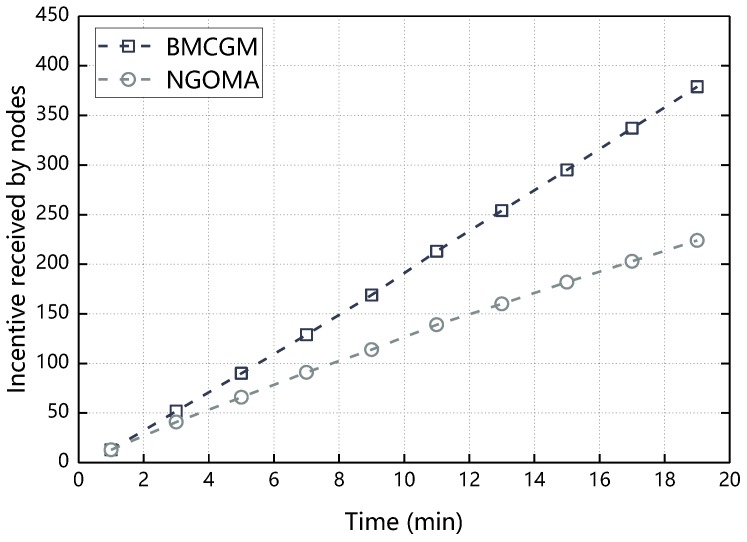
Incentive received by nodes with respect to time.

Figure 10 compares the difference between the BMCGM and NGOMA in the aspect of incentive received by nodes. As can be seen from Figure 10, as the experimental running time increases, incentive received by nodes increase in BMCGM and NGOMA with the nodes participate in cooperation. While node’s incentive in BMCGM is greater than the node’s incentive in NGOMA, this is because in NGOMA, only the influence of Γ on the choice of node behavior is considered and the node only cooperates in the case of maximizing its own Γ. In the BMCGM, the reciprocal altruistic of the node is considered. Compared to the traditional message cooperation transmission mechanism, nodes are more likely to achieve cooperative behavior, so nodes in BMCGM received more incentive than NGOMA.

#### 5.4.2. Incentive Received Ratio with Respect to Time

**Figure 11:**
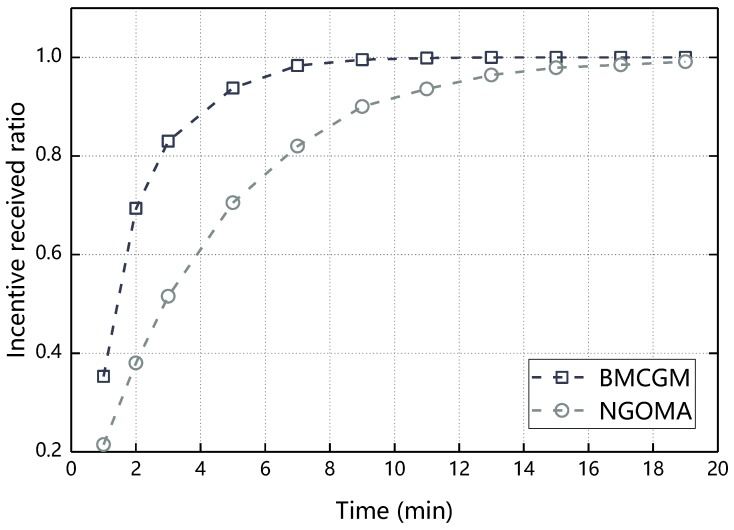
Incentive received ratio with respect to time.

Figure 11 compares the difference between the BMCGM and NGOMA in the aspect of incentive received ratio. It can be seen from Figure 9 that incentive received ratios of the two algorithms increase with time and the growth in the early part of the experiment is faster and the latter part increases slowly and finally reaches 1. This is because, assume that there are n nodes in the network, the nodes randomly send messages to each other and the message is sent from the source node to the destination node through one or more relay nodes. Suppose that there are a nodes participate in the message cooperative transmission after the i^th^ random interaction, then the proportion of nodes that have not obtained incentive after the interaction is (n−a)/n. When the (i+1)^th^ interaction starts, it is obvious that if the value of a is larger, the proportion of nodes that have not participated in the cooperation before is smaller, so the growth in the early part of the experiment is faster and the latter part increases slowly. At the same time, it can be seen from Figure 11, BMCGM’s incentive received ratio grows faster than the NGOMA and reaches 1 in 11 min. This is because, in the case of considering the reciprocal altruistic of nodes, the node is more willing to cooperate.

#### 5.4.3. Delay Ratio with Respect to Time

**Figure 12:**
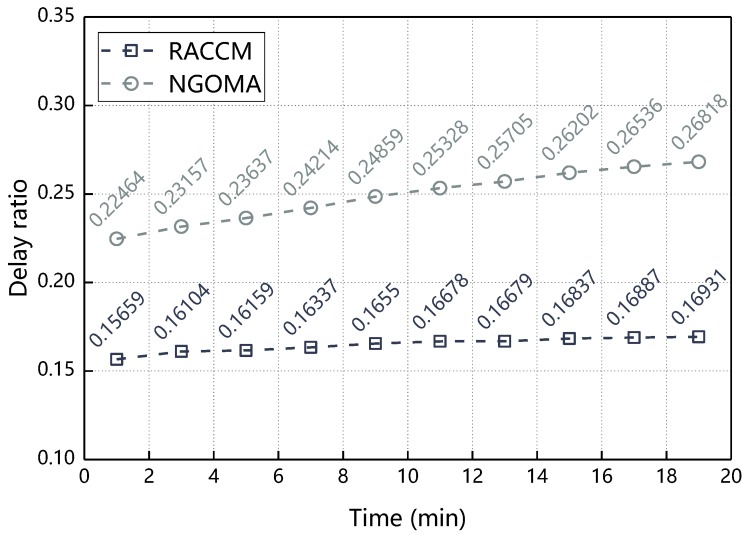
Delay ratio with respect to time.

Delay refers to the time taken for the message to be sent from the source node to the destination node. The delay ratio in this section refers to the ratio of delay to the current running time. Figure 12 comparing the performance difference between BMCGM and NGOMA in delay ratio. As can be seen from the figure, both the BMCGM and NGOMA delay ratio increase with time but the BMCGM delay ratio is smaller than the NGOMA delay ratio, this is because in BMCGM, Π of nodes with altruistic reciprocal attributes is higher than the self-interested nodes assumed in the traditional message cooperative transmission mechanism. In NGOMA, when the source node and the node around the destination node cannot maximize their own incentive by cooperating transmit messages, they will not choose cooperation. In BMCGM, the node with the altruistic reciprocal attribute will be affected by Φ and the cooperation behavior will be selected. The cooperative behavior that cannot be achieved in NGOMA can be reached in BMCGM, thus reduces the delay ratio, so BMCGM has a lower delay ratio than NGOMA.

#### 5.4.4. Transmitted Packet Ratio with Respect to Time

**Figure 13:**
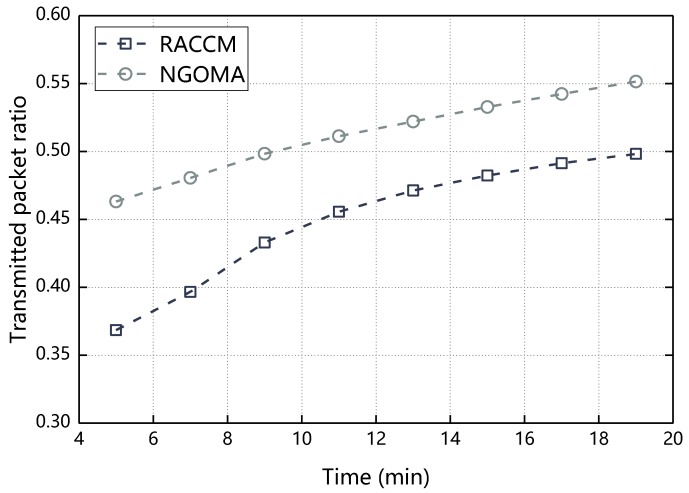
Transmitted packet ratio with respect to time.

The transmitted packet count refers to the number of messages sent between the source node and the destination node. Therefore, when the number of messages between the source node and the destination node increases, the transmitted packet count increases accordingly. Figure 13 compares the performance difference between BMCGM and NGOMA in the aspect of transmitted packet ratio. It can be seen from the figure that the transmitted packet ratio of BMCGM and NGOMA increases with time and compared with NGOMA, BMCGM has lower transmitted packet ratio, that is because in NGOMA, when the source node needs to send a message to the destination node through one or more relay nodes, if these nodes cannot maximize their own aggregation through cooperative message transmission, these nodes will not cooperate, at this time, the source node needs to wait for the message to be directly transmitted to the destination node. when the cause such as channel congestion occurs, the destination node cannot receive the message or the source node cannot receive the acknowledge message of the destination node and the source node attempts to retransmit the message to the destination node, the transmitted packet count will increase and the transmitted packet ratio will increase. In BMCGM, the node is more willing to cooperate because of the reciprocal altruistic of the node, so the transmitted packet ratio is lower.

### 5.5. The Impact of δ on BMCGM

This section analyzes the effects of reciprocal altruistic coefficient δ on BMCGM from four aspects: incentive received by nodes, average incentive received ratio, average delay ratio and average transmitted packet ratio.

**Figure 14:**
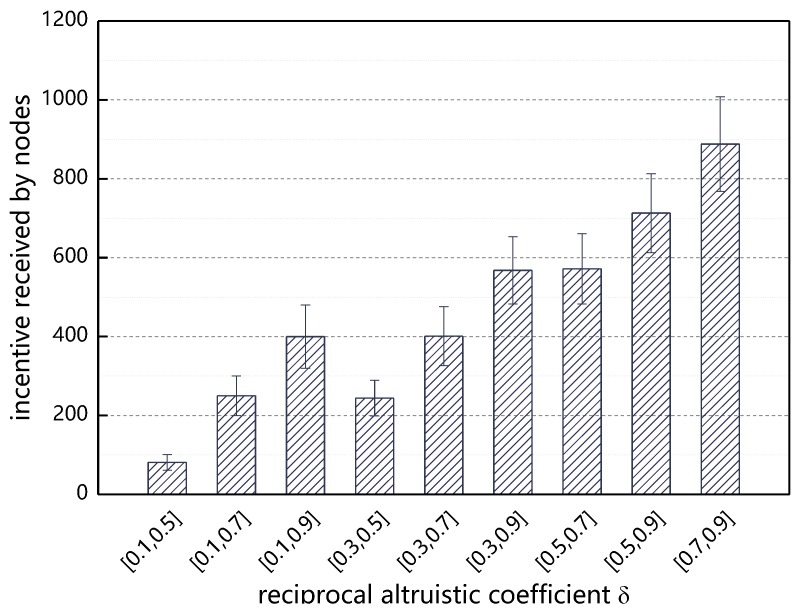
Incentive received by nodes with respect to δ.

Figure 14 Shows the effect of different value intervals of δ on the total utility of the nodes after 20 min of experimental operation. It can be seen from the figure that when δ is gradually increasing from [0.1, 0.5] to [0.1, 0.9], or gradually increasing from [0.3, 0.5] to [0.3, 0.9] and gradually increasing from [0.5, 0.9] to [0.7, 0.9], the total utility of the node is increasing. This result shows that when the average reciprocal altruistic property of the node increases, the final utility of the node will also increase. At the same time, it can be seen from the figure that when the average level of reciprocal altruistic is the same, that is, when the node’s reciprocal altruistic attribute value interval is [0.1, 0.7] or [0.3, 0.5], the final utility of the node is similar.

Table 4 shows the influence of the reciprocal altruistic δ in 3 aspects: the average incentive received ratio, the average delay ratio and the average transmitted packet ratio. When δ∈ [0.0, 0.0], it means that the reciprocal altruistic attribute of the node is not considered at this time, that is, traditional message cooperation transmission mechanism. It can be seen from the table that the value range of δ gradually increases from [0.1, 0.5] to [0.1, 0.9], or from [0.3, 0.5] to [0.3, 0.9] and from [0.5, 0.7] to [0.7, 0.9], BMCGM’s average incentive received ratio gradually increases, while the average delay ratio and average transmitted packet ratio are gradually reduced. It fully demonstrates that the increment of δ can promote the cooperative behavior between nodes and improve the cooperation rate of nodes, while reducing the delay of message cooperation transmission and average transmitted packet ratio. Compared with the traditional message cooperation transmission mechanism. It means δ∈ [0.0, 0.0], even when the average level of reciprocal altruistic of the node is low (δ∈ [0.1, 0.5], $\bar{\delta }=0.4$), the performance of BMCGM is still better than the traditional message cooperation communication mechanism.

## 6. Conclusions

In this paper, based on the inspiration of behavioral economics, we use reciprocal altruistic in the cooperation transmission mechanism and construct a new message transmission cooperation guarantee mechanism named BMCGM. We construct a cooperative willingness function based on reciprocal altruistic which can reflect the friendly of other nodes. We design a reciprocal utility function based on cooperative willingness function, where the utility of a node is determined by the combination of economical utility and reciprocal utility. In BMCGM, the nodes’ behavior is analyzed based on reciprocal altruistic and nodes’ cooperation behavior are promoted. Simulation results show that BMCGM reduces message transmission delay by at least 30.3% and transmitted packet ratio by at least 10% compared with the recent representative cooperation transmission mechanism NGOMA.

## Figures and Tables

**Table 1:** The mapping of vampire bat experiment and incentive mechanism.

	Participants	Events	Decisions
Vampire bat experiment (A)	Vampire bat	Some bats are fasted	Bats getting food feedback to fasted bats
Vampire bat experiment (B)	Vampire bat	Bats are fasted in rotation, including bats without back-feeding behavior	Bats that are not fasted are fed back to bats that have been back-feeding
Traditional cooperation mechanism	Vehicle nodes	Nodes request help for forwarding messages from surrounding node	The surrounding nodes choose to cooperate based on a utility value $U=U_{p}$ which can maximize their own incentives.
The proposed BMCGM	Vehicle nodes	Nodes request help for forwarding messages from surrounding node	Due to the reciprocal altruistic of the node, there is a reciprocal utility caused by reciprocal cooperation which makes $U^{\prime }=U_{p}^{\prime }+U_{c}>U$, the node will choose to cooperate
Commonality	Ability to judge gains and losses	There are participants who need help/cooperation	Whether other participants make decisions to help/cooperate

U and $U^{\prime }$ indicate the total utility of the node, $U_{p}$ and $U_{p}^{\prime }$ indicate the economic utility of the node. $U_{c}$ indicates the reciprocal utility of the node.

**Table 2:** The Notion used in Behavior Economics-based Message Transmission Cooperation Guarantee Mechanism (BMCGM).

Parameter	Definition
G	Network topology
A, $A_{i}$	Node, node i
Γ, $\Gamma_{i}$	Economic utility, node i’s economic utility
Π, $\Pi_{ij}$	The cooperative willingness, The cooperative willingness that node i to node j
δ, $\delta_{i}$	The level of reciprocal altruistic, The level of node i’s reciprocal altruistic
Φ, $\Phi_{i}$	Reciprocal utility, node i’s reciprocal utility
γ	Reciprocal altruistic cooperation influence coefficient
μ	Cost coefficient
C, $C_{i}$	Cost, node i’s cost
Λ, $\Lambda_{i}$	Utility, node i’s utility
s, $s^{c}$, $s_{i}^{c}$, $s_{ij}$	Strategy, node’s current strategy, node i’s current strategy, strategy that node i choose to cooperate with node j
$G_{s_{i}^{c},-s_{{}^{i}}^{c}}$	The network topology while node i and other nodes remain their current strategy
$G_{s_{i}^{c},-s_{{}^{i}}^{c}}+s_{ij}$	The network topology while node i choose $s_{ij}$ and other nodes remain their current strategy

**Table 3:** Simulation parameters.

Parameters	Value or Range
Number of lanes	2
Number of nodes	23
The length of lanes	2000 m
The width of lanes	10 m
Maximum node speed	60 m/s
Minimum node speed	10 m/s
Node’s transmission range	300 m
Cost coefficient μ	0.1, 0.15, 0.155, 0.160, 0.165, 0.170, 0.175, 0.2
Cooperating influence coefficient γ	0.1, 0.15, 0.155, 0.160, 0.165, 0.170, 0.175, 0.25
Reciprocal utility proportion coefficient β	(0, 0.9)
reciprocal altruistic coefficient δ	[0, 0], [0.1, 0.5], [0.1, 0.7], [0.1, 0.9], [0.3, 0.5], [0.3, 0.7], [0.3, 0.9], [0.5, 0.9], [0.7, 0.9]

**Table 4:** The impact of δ on the performance of BMCGM.

$[\delta^{\min},\;\delta^{\max}]$	Average Incentive Received Ratio	Average Delay Ratio	Average Transmitted Packet Ratio
[0, 0]	0.822500	0.236230	0.500978
[0.1, 0.5]	0.930196	0.176718	0.433636
[0.1, 0.7]	0.930783	0.171194	0.431537
[0.1, 0.9]	0.930816	0.165311	0.430759
[0.3, 0.5]	0.930483	0.171045	0.431161
[0.3, 0.7]	0.930535	0.165386	0.431021
[0.3, 0.9]	0.930811	0.157926	0.429031
[0.5, 0.7]	0.931072	0.157916	0.425436
[0.5, 0.9]	0.931841	0.146634	0.357593
[0.7, 0.9]	0.932115	0.135721	0.356618
